# Development of New Targeted Inulin Complex Nanoaggregates for siRNA Delivery in Antitumor Therapy

**DOI:** 10.3390/molecules26061713

**Published:** 2021-03-19

**Authors:** Gennara Cavallaro, Carla Sardo, Emanuela Fabiola Craparo, Gaetano Giammona

**Affiliations:** 1 Lab of Biocompatible Polymers, Department of Biological, Chemical and Pharmaceutical Sciences and Technologies (STEBICEF), University of Palermo, Via Archirafi 32, 90123 Palermo, Italy; csardo@unisa.it (C.S.); emanuela.craparo@unipa.it (E.F.C.); gaetano.giammona@unipa.it (G.G.); 2 Department of Pharmacy, University of Salerno, Via Giovanni Paolo II, 132, 84084 Fisciano, Italy

**Keywords:** siRNA delivery, inulin, PEG, EGF, targeting, tumour

## Abstract

Here, a novel strategy of formulating efficient polymeric carriers based on the already described INU-IMI-DETA for gene material whose structural, functional, and biological properties can be modulated and improved was successfully investigated. In particular, two novel derivatives of INU-IMI-DETA graft copolymer were synthesized by chemical functionalisation with epidermal growth factor (EGF) or polyethylenglycol (PEG), named INU-IMI-DETA-EGF and INU-IMI-DETA-PEG, respectively, in order to improve the performance of already described “inulin complex nanoaggregates” (ICONs). The latter were thus prepared by appropriately mixing the two copolymers, by varying each component from 0 to 100 wt% on the total mixture, named EP-ICONs. It was seen that the ability of the INU-IMI-DETA-EGF/INU-IMI-DETA-PEG polymeric mixture to complex siGL3 increases with the increase in the EGF-based component in the EP-ICONs and, for each sample, with the increase in the copolymer:siRNA weight ratio (R). On the other hand, the susceptibility of loaded siRNA towards RNase decreases with the increase in the pegylated component in the polymeric mixture. At all R values, the average size and the zeta potential values are suitable for escaping from the RES system and suitable for prolonged intravenous circulation. By means of biological characterisation, it was shown that MCF-7 cells are able to internalize mainly the siRNA-loaded into EGF-decorated complexes, with a significant difference from ICONs, confirming its targeting function. The targeting effect of EGF on EP-ICONs was further demonstrated by a competitive cell uptake study, i.e., after cell pre-treatment with EGF. Finally, it was shown that the complexes containing both EGF and PEG are capable of promoting the internalisation and therefore the transfection of siSUR, a siRNA acting against surviving mRNA, and to increase the sensitivity to an anticancer agent, such as doxorubicin.

## 1. Introduction

In recent years, inulin (INU) is being increasingly used as a polymer backbone to construct advanced functional materials for drug and nucleic acid delivery due to its biocompatibility and biodegradability [1,2]. Many examples of INU-based copolymers have already been obtained by conjugation of molecules such as α-tocopherol [2], ceramide [3], and targeting agents such as biotin [4], and have been proposed for biomedical applications.

Epidermal growth factor receptor (EGFR) is a transmembrane receptor that binds ligands from the endogenous epidermal growth factor (EGF) family, which results in receptor internalisation primarily via clathrin-mediated pathways [5]. As EGFR is implicated in cancer progression and poor prognosis, several anti-EGFR treatment strategies have been clinically approved in recent years [5]. Thanks to its high binding affinity to the EGFR receptor, several EGFR-binding ligands, such as EGF or its functional fragments, were proposed as decorating molecules for anti-tumour drug-loaded nanoparticle surfaces to achieve tumour cell-specific delivery [6]. The low molecular weight of EGF makes it an attractive alternative to antibodies to decorate the nanoparticle surfaces with a proper density.

Past experience of other research groups fosters the validity of the approach: Tseng and co-workers reported that aerosol administration of EGF-decorated gelatine nanoparticles in mice produced in 24 h a specific accumulation in lung adenocarcinoma compared with unmodified nanoparticles [7]. Wang et al. reported that EGF-modified nanoparticles based on methoxy polyethylene glycol-polylactic-co-glycolic acid-polylysine (PEG-PLGA-PLL) block copolymer were able to improve either the systemic toxicity aspect or therapeutic efficiency of the loaded cisplatin [8]. Fonge et al. made indium-loaded EGF-conjugated polymeric micelles for targeted auger electron radiotherapy, which were able to cause 100% breast cancer cell death in vitro [9].

Small interfering RNAs (siRNAs) are an emerging class of nucleic acids that mediate gene silencing of a target protein by disrupting messenger RNAs in an efficient and sequence-specific manner [10]. Their most important potential application is the knockdown of genes responsible for tumorigenesis, proliferation, survival, and drug resistance of tumour cells [11]. However, clinical siRNA translation still requires efforts in order to protect siRNA from plasmatic and tissue RNase, elude the reticuloendothelial system (RES), and to improve the overcoming of biological barriers such as endothelial and tissue cellular membranes [12]. Thus, the identification of efficient and highly biocompatible, as well as cost-effective, materials is crucial for the development of effective siRNA delivery carriers. Moreover, the possibility of having available materials whose structural, functional, and biological properties can be properly modulated and improved as a function of the specific siRNA could be an additional advantage to pursue. Recently, our group has focused on the development of inulin (INU) derivatives for gene therapy in order to overcome the cellular toxicity associated with polycations [12]. In particular, we described the synthesis of INU-based polycations obtained by grafting to hydroxyls of fructose repeating units linear oligoamines, such as spermine (Spm), diethylenetriamine (DETA), and ethylenediamine (EDA), to obtain INU-Spm, INU-DETA, and INU-EDA polycations, respectively, that are charged at physiological pH [12,13]. The in vitro studies on INU-DETA silencing effects encouraged the rational design and synthesis of a unique pH-responsive polycation, structurally derived from INU-DETA, the INU-g-imidazole-g-DETA (INU-IMI-DETA), able to complex a higher amount of siRNA to obtain “inulin complex nanoaggregates” (ICONs) [13]. It has been demonstrated that the obtained ICONs possessed slight surface charge and suitable size for parenteral administration, and were able to overcome specific cellular and subcellular barriers. Indeed, once internalised, these were able to escape from the endosomal compartment due to the imidazole proton sponge effect [13] and 1,2-diaminoethane’s destabilising effect [14] on endosomal membranes. At this point, it was stimulating to improve this very promising carrier system by giving new specific features such as the ability to circulate undisturbed in the bloodstream and the possibility of accumulating in cancer cells interacting with them in a specific way.

Therefore, in this paper, the ICONs’ performance was f urther improved by conjugating on INU-IMI-DETA a specific active targeting ligand, in order to increase the specific cell uptake and decrease it in off-target cells and organs, allowing their in vivo applicability. In particular, we successfully conjugated epithelial growth factor (EGF) on INU-IMI-DETA to obtain the INU-IMI-DETA-EGF graft copolymer. Moreover, in order to improve the stability and circulation time of the nanoaggregates, a pegylated derivative of INU-IMI-DETA was synthesized by conjugation of polyethylenglycol (PEG) in the INU-IMI-DETA copolymer structure. The strategy of synthesising two different copolymers and not a single copolymer with all the functions present in its structure was pursued in order to modulate ad hoc the composition of the resulting polymeric carrier according to the gene material to be conveyed. Thus, nanoaggregates with tuneable composition in terms of PEG and EGF were formulated by mixing different amounts of INU-IMI-DETA-EGF and INU-IMI-DETA-PEG (EGF and PEG containing inulin complex nanoaggregates—EP-ICONs), to give the renowned PEG-related properties to the final siRNA delivery system, such as colloidal stability and long circulation with minimized recognition by RES [12].

EP-ICONs were produced by complexing a siRNA directed against survivin (siSUR). This, a member of the inhibitors of apoptosis (IAP) family upregulated in the majority of breast cancers, plays an important role in regulating cell division and suppressing apoptosis, enabling it to be a potential target gene for cancer therapy [15,16]. Recently, it was demonstrated that targeting survivin in experimental models improves survival [17], with these proteins being effectors of doxorubicin (Doxo) resistance in breast cancer [18]. By means of in vitro experiments, we demonstrated the importance of both complexation or chemical composition of our aggregates on the intracellular uptake and chemosensitisation to Doxo.

## 2. Results and Discussion

### 2.1. Synthesis and Characterisation of INU-IMI-DETA-EGF and INU-IMI-DETA-PEG Copolymers

In the present study, human recombinant EGF (EGF) was directly conjugated on the previously reported INU-g-imidazole-g-diethylenetriamine (INU-IMI-DETA) graft copolymer, [13] obtaining the INU-IMI-DETA-EGF graft copolymer, in order to improve its performance and to realise targeted “inulin complex nanoaggregates” (ICONs) for siRNA delivery.

**Figure 1:**
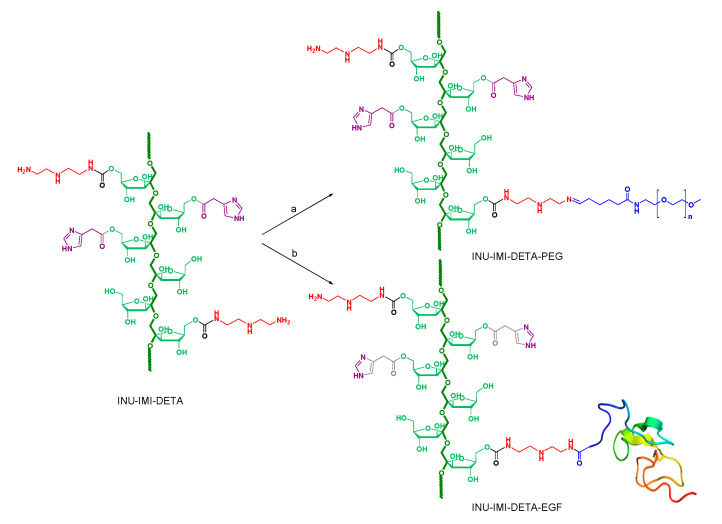
Schematic representation of INU-IMI-DETA-EGF synthesis. (**a**) PEG_2000_CHO, water, pH5, r.t. overnight; (**b**) EDCI, NHS, hEGF, water, r.t. 6 h.

In particular, we synthesized INU-IMI-DETA-EGF through a coupling reaction between primary amine groups of pending DETA molecules and carboxyl groups of hEGF by using *N*-(3-dimethylaminopropyl)-*N*′-ethyl carbodiimide hydrochloride (EDCI)/N-hydroxysuccinimide (NHS) as coupling reagents forming amide bonds (Figure 1b). The EDCI/NHS coupling reaction being very mild, it was easily completed within 6 h at room temperature.

The occurrence of EGF conjugation on INU-IMI-DETA was evaluated by FT-IR analysis and Thannauser assay on INU-IMI-DETA-EGF graft copolymer [19].

The FT-IR spectrum of INU-IMI-DETA-EGF (Figure S1) showed the presence of signals at 1645 and 1538 cm^−1^ attributable to EGF amide I and amide II vibrations, respectively (that are absent in the INU-IMI-DETA spectrum), that qualitatively indicated the occurrence of the conjugation reaction.

EGF quantification was carried out by determining the amount of thiols in EGF (related to the 6 cysteine residues) by Thannhauser assay [19], which gives a degree of functionalisation in EGF of 1.53 ± 0.17 µmol/mg% (corresponding to 0.50 ± 0.03 mmol% of fructose repeating units on INU backbone).

With the aim to further improve the in vivo performance of the ICONs, polyethylene glycol (PEG) was also conjugated to the INU-IMI-DETA graft copolymer, due to its unique properties to confer biocompatibility and to increase the solubility of colloidal systems [12,20,21]. Improvements in performance also arise from the substantial changes in the biodistribution, reduction in opsonisation, and increase in blood circulation time of these carriers, which could result in an increased tumour accumulation [12,22].

The choice to synthesize two different INU derivatives, the pegylated and the EGF-decorated ones, arises from two considerations: on the one hand, since both the EGF and the PEG are grafted by reaction with the DETA terminal amino groups, if it were sequentially done on the same polymeric chains, it would lead to a lower capability to complex siRNA of the resulting polymer with respect to the INU-IMI-DETA due to the reduction in the total free amine content. On the other hand, the use of two different copolymers, INU-IMI-DETA-EGF and INU-IMI-DETA-PEG, which are decorated with EGF and PEG ligands, respectively, allows us to properly modulate the composition of the resulting ICONs complexes, thus giving the possibility to select the best performing system for gene therapy.

In detail, the pegylated INU-IMI-DETA derivative was synthesized by alkylimino-de-oxo-bisubstitution of a monofunctional aldehydic derivative of PEG (2 kDa molecular weight), with DETA primary amino groups pending from INU backbone, thus forming a Schiff base (Figure 1a). The derivatisation degree in PEG (DD_PEG_) was determined by ^1^H-NMR by comparing the integral of PEG protons with the integral relative to protons of the INU backbone (see Figure S2 in Supplementary Materials), and resulted to be equal to 3.29 ± 0.28 mol% of fructose repeating units on the INU backbone. A comparison between representative ^1^H-NMR spectra of INU-IMI-DETA-EGF, INU-IMI-DETA-PEG, and INU-IMI-DETA can be also found in Supplementary Materials Figure S2.

Both INU-IMI-DETA-EGF or INU-IMI-DETA-PEG graft copolymers showed pH sensitive properties as INU-IMI-DETA, being almost positively charged, therefore freely soluble at pH values lower than 5 (data not shown). At pH 7.4, due to the charge reduction on the polymeric backbone, both copolymers showed high hemocompatibility, as demonstrated by the absence of agglutination and haemolysis phenomena on human red blood cells (hRBC) in the chosen experimental conditions (Figure S3 in Supporting Information). Moreover, the absence of effects of both copolymers on the viability of 16HBE, MCF-7 living cells was demonstrated until 72 h incubation (Figure S4 in Supporting Information).

### 2.2. Preparation and Characterisation of EP-ICONs

Once obtained and properly characterised, INU-IMI-DETA-EGF and INU-IMI-DETA-PEG graft copolymers were used to produce ICONs with siRNA by following the same preparation method described previously for INU-IMI-DETA [13]. In particular, a proper volume of a water acidic copolymer solution (containing a mixture of INU-IMI-DETA-EGF and INU-IMI-DETA-PEG at a weight ratio equal to 90:10, 70:30 or 50:50) was mixed with the same volume of a siGL3 aqueous solution in order to realise defined polymer to siRNA weight ratios (R) ranging between 5 and 50. At this stage, thanks to extensive protonation of IMI and DETA moieties, the copolymers are freely soluble and able to strongly interact with siRNA via electrostatic interaction to form polyplexes. Afterwards, the copolymer/siRNA complexes were added into DPBS buffer (pH 7.4) to produce “EGF/PEG functionalised INU-IMI-DETA/siRNA complex nanoaggregates (EP-ICONs). Control formulations were obtained by using as copolymer INU-IMI-DETA-EGF or INU-IMI-DETA-PEG alone to obtain polyplexes with siGL3, respectively indicated as E-ICONs and P-ICONs. The composition of each sample (EP-ICONs, E-ICONs, and P-ICONs) in terms of µmol% of PEG and/or EGF ligands per mg of copolymer blend is reported in Table 1.

**Figure 2:**
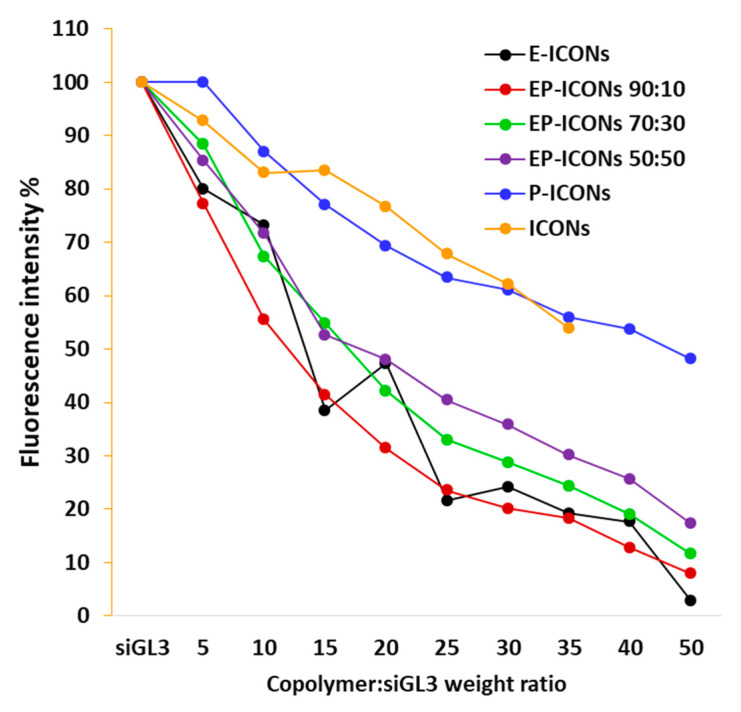
Fluorescence intensity % of ethidium bromide (EtBr) in the presence of unloaded siGL3 after EP-ICONs, P-ICONs, E-ICONs, and ICONs formation at R ranging between 5 and 50.

In order to evaluate the encapsulation efficiency (EE%) of siRNA in each sample as a function of either copolymer composition of EP-ICONs or the copolymer/siRNA weight ratio (R), an ethidium bromide (EtBr) exclusion assay was carried out. In particular, the amount of unloaded siRNA was determined after the formation of all nanoaggregates at various compositions, such as EP-ICONs at 50:50, 70:30, and 90:10 INU-IMI-DETA-EGF:INU-IMI-DETA-PEG weight ratios, P-ICONs, E-ICONs and ICONs (for comparison), and at copolymer:siRNA weight ratios (R) ranging between 5 and 50. The results are reported in Figure 2.

The results in terms of unloaded siRNA highlight that EE% increases as INU-IMU-DETA-EGF graft copolymer increases in the composition (reaching 98% of siRNA encapsulation efficiency with E-ICONs at R50), while P-ICONs showed an EE% comparable to ICONs, evidencing that the presence of EGF improves the interaction of the copolymer with siRNAs. These findings will open up new perspectives on the design of polyplexes in which the chosen targeting moiety contributes to drug loading.

The capability of the complexing copolymer to protect the genetic material from degradation phenomena in biological fluids (i.e., by nuclease) is also an important aspect to evaluate for the development of novel polycations. For this reason, a stability study was carried out in the presence of RNase.

**Figure 3:**
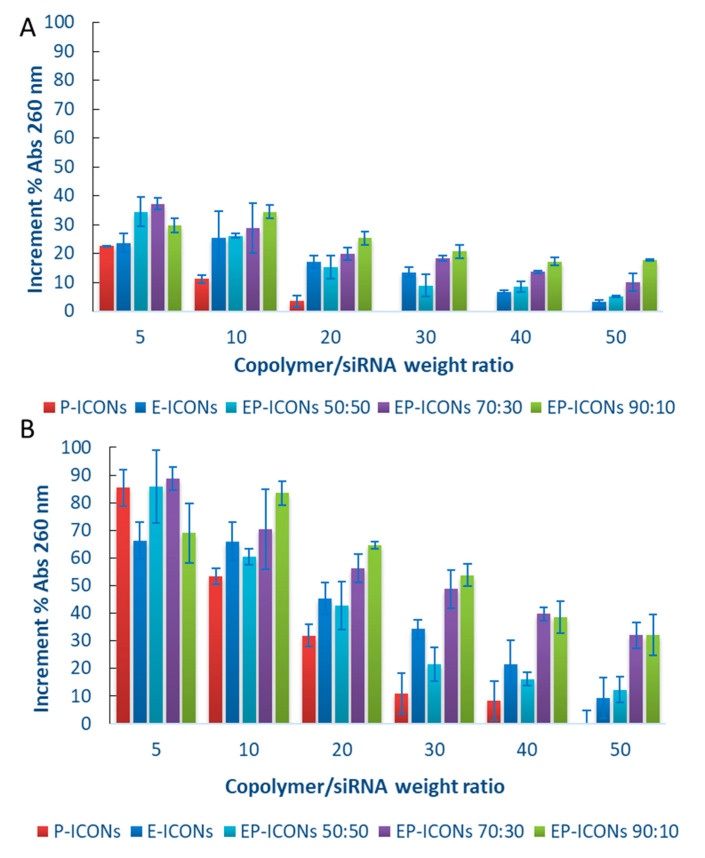
RNase protection assay. (**A**) 4 h, (**B**) 24 h.

Figure 3A,B show the hyperchromic effect, i.e., the increase in absorbance at λ = 260 nm when nucleic acids are degraded, obtained after 4 and 24 h incubation, respectively, of EP-ICONs, E-ICONs, and P-ICONs in the presence of RNase.

It is evident that siRNA stability increases as the INU-IMI-DETA-PEG graft copolymer in the EP-ICONs composition increases, the obtained absorbance value being the lowest with P-ICONs. This effect reasonably could arise from the formation of a PEG hydrophilic shell in the ICONs, whose density grows with the amount of INU-IMI-DETA-PEG in the mixture, leading to a progressively higher shielding of the system from the enzyme approach. Therefore, although the complexation capability was higher for E-ICONs, the presence of a certain amount of PEG increased the cargo stability against enzymatic degradation. However, at the highest R, a significant protection was found even for the systems with the lowest PEG content (EP-ICONs 90:10, R50, 24 h in Figure 3B), which caused 30% siRNA degradation. In Figure S5A–E of the Supporting Information section, the degradation profiles of siRNA loaded in all the tested samples (R5–R50) are also reported in detail as a function of incubation time with RNase.

Therefore, a fair compromise between the INU-IMI-DETA-EGF and INU-IMI-DETA-PEG graft copolymers containing two different ligands with specific functionalities, one EGF with potentially directing properties, and the other the PEG with shielding properties, is desirable.

**Figure 4:**
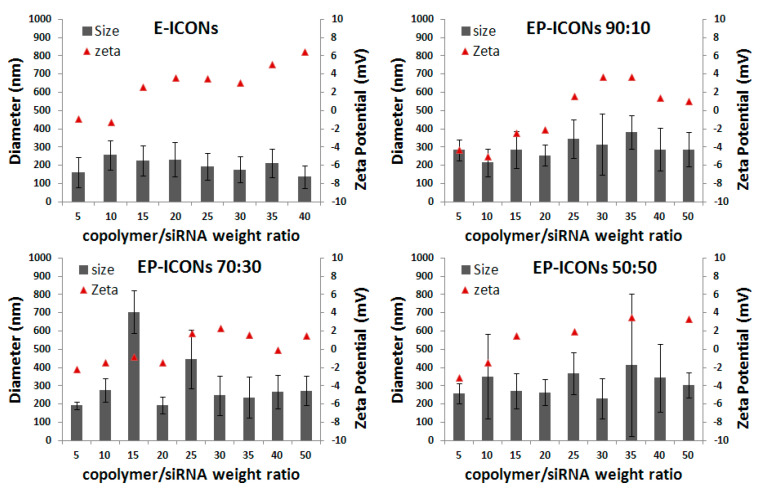
Size and ζ potential values of E-ICONs and EP-ICONs (obtained at INU-IMI-DETA-EGF: INU-IMI-DETA-PEG copolymer weight ratio equal to 90:10, 70:30, 50:50) at copolymer/siRNA weight ratios (R) ranging between 5 and 50.

Physicochemical properties such as particle size, surface charge, and the composition of nanoaggregates are known to have significant effects on cellular uptake and biodistribution [23,24]. Multiple findings support the indication that nanoparticles with a slightly negatively charged surface can reduce the undesirable clearance by RES and improve the blood compatibility, thus delivering drugs more efficiently to the site of action [8,25] Therefore, approaches for escaping the phagocyte uptake included maintaining a discrete particle size and keeping the ζ potential below 15 mV. For this reason, both mean size and zeta potential of EP-ICONs, as well as E-ICONs and P-ICONs, were evaluated in DPBS by dynamic light scattering (DLS). Obtained mean size and ζ potential values for E-ICONs and EP-ICONs are reported in Figure 4. The values obtained by P-ICON characterisation are not shown for simplification, being superimposable to those obtained with EP-ICONs 90:10.

All samples showed means size ranging between 100 and 400 nm, without effects relating to R, although polydispersity increases as the INU-IMU-DETA-EGF amount decreased in the copolymer mixture used to produce aggregates with siRNA. ζ potential of all EGF-based ICONs slightly increased with increasing R, but never reached values higher than 6 mV. Therefore, the obtained systems showed mean size and surface charge suitable to be dispersed in a physiological medium, such as DPBS, for parenteral administration, accordingly to that reported before for ICONs, demonstrating that the introduction of EGF and PEG ligands keep the colloidal stability.

### 2.3. In Vitro Characterisation: Targeting and Chemosensitisation Studies

Once it was demonstrated that EP-ICONs possess suitable properties in terms of mean size, zeta potential, high capability to entrap siRNA and protect it from RNase activity, which can be modulated by varying the copolymer composition in terms of INU-IMU-DETA-EGF/INU-IMU-DETA-PEG weight ratio, biological experiments were carried out on human breast cancer cells (MCF-7) to evaluate their potential to be used as siRNA carriers.

**Figure 5:**
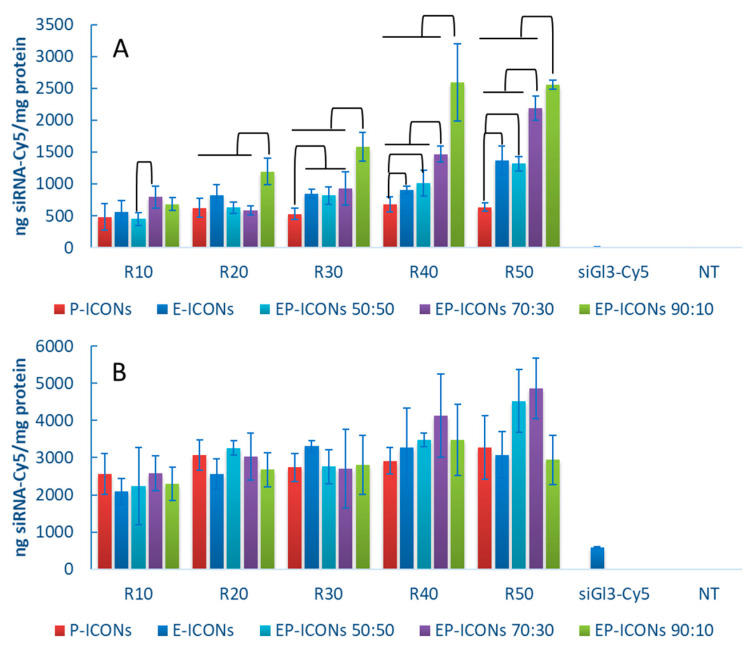
Quantitative uptake of siGL3-Cy5 in MCF-7 cells after 4 h (**A**) and 24 h (**B**) incubation with EP-ICONs at copolymer:siRNA weight ratios (R) comprised between 10 and 50. Results were compared with each other and with P-ICONs, E-ICONs, naked siRNA (siGL3-Cy5) and untreated cells (NT). Connections represent statistical differences with *p* < 0.05.

First, in order to investigate the capability of our nanoaggregates to allow the cell internalisation of a loaded siRNA, an in vitro cell uptake study was carried out by EP-ICONs loaded with siGL3-Cy5. In particular, the amount of the fluorescent siRNA was quantified in MCF-7 cell lysates after 4 and 24 h of incubation with siGL3-Cy5-loaded EP-ICONs at various INU-IMI-DETA-EGF/INU-IMI-DETA-PEG weight ratios. As can be seen from Figure 5A, after 4 h incubation, an increasing uptake of siGL3-Cy5 was evidenced as the INU-IMI-DETA-EGF weight percentage in EP-ICONs increases. In this case, the data also support the hypothesis that balance of the two components, INU-IMI-DETA-EGF and INU-IMI-DETA-PEG can play the major role in determining the effectiveness of the delivery system, giving an appropriate equilibrium between the capacity of siRNA retention/release and between colloidal stability/uptake. However, low cell internalisation of siGL3-Cy5 entrapped into E-ICONs (comparable to those obtained with P-ICONs) supports the hypothesis that EGF could be directly involved in the interaction with siRNA and hence is not fully available for receptor binding and receptor-mediated uptake promotion when employed without the pegylated copolymer.

However, after 24 h incubation (see Figure 5B), the siGL3-Cy5 cell uptake is comparable for all the investigated samples, confirming that the siRNA complexation with polycations into polyplexes allow the cell internalisation, which is forbidden to naked siRNA.

**Figure 6:**
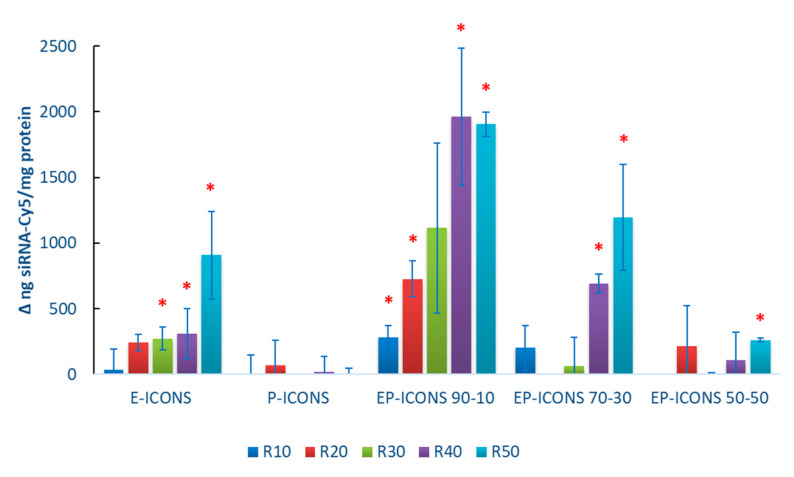
EGF competitive cellular uptake studies. Data are expressed as the differences between uptake without EGF pre-incubation and EGF competitive uptake. Statistically significant values are indicated with asterisks (* *p* < 0.05).

To confirm the involvement of EGF in cellular uptake, a competitive cellular uptake study was carried out by pre-incubating MCF-7 cells with free EGF. In Figure 6, differences between the siRNA-Cy5 amounts taken up by MCF-7 cells treated with siRNA-Cy5 loaded EP-ICONs (at R between 10–50) after pre-incubation in the absence or in the presence of free EGF are reported.

As can be seen, the pre-incubation of cells with free EGF does not affect the internalisation of siRNA loaded into P-ICONs. In the presence of INU-IMI-DETA-EGF in the copolymer composition, a significant reduction in the siRNA internalisation is evidenced, that could indicate a targeting effect of EGF. In particular, as the INU-IMI-DETA-EGF amount increases in EP-ICONs (from INU-IMI-DETA-EGF/INU-IMI-DETA-PEG copolymer weight ratios equal to 50:50 to 90:10), the difference became significant as R increases. This trend perfectly fits with the hypothesis that EGF-mediated uptake acts as a targeting ligand.

Therefore, the strategy to modulate the polycation composition by blending INU-IMI-DETA-EGF and INU-IMI-DETA-PEG copolymers has proven to be effective in improving the cell internalisation of siRNA, compared to the use of INU-IMI-DETA-EGF copolymer alone. In fact, E-ICONs determined a significant uptake reduction only from R20 to R50; while reducing the amount of INU-IMI-DETA-EGF in the EP-ICONs by about 10 wt%, a significant uptake reduction is obtained at all the tested R after cell pre-incubation with free EGF, which means EGFR saturation.

Once it was demonstrated that our EP-ICONs are able to carry inside cells the loaded genetic material and that this process is mediated by the EGF, their potential application in anti-tumour therapy was evaluated [11].

In this experiment, a siRNA against survivin mRNA was chosen as a potential therapeutic target to treat breast cancer [15,17]. siSUR was chosen as cargo, as survivin is a member of the inhibitor of apoptosis proteins (IAP) family and plays a positive promotion role in tumour cells proliferation and in inhibiting cell apoptosis [16].

**Figure 7:**
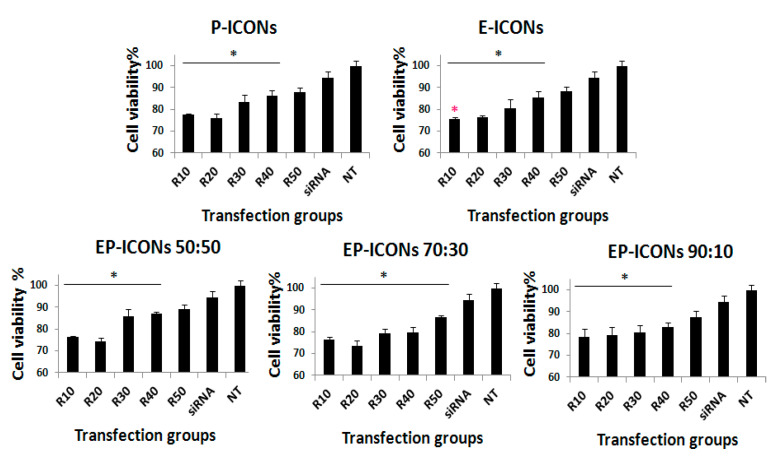
Proliferation of MCF-7 cells after 72 h incubation with E-ICONs, P-ICONs, or EP-ICONs containing survivin targeted siRNA. * represents *p* < 0.05 with respect to siRNA naked treated cells. A red asterisk on E-ICONs R10 represents significant reduction when compared with P-ICONs at the same weight ratio.

Therefore, EP-ICONs loaded with siSUR were prepared, and their inhibition effect of MCF-7 viability by the MTS assay. The results reported in Figure 7 show that, after 72 h incubation, there was no difference in cell viability between naked siSUR (200 nM) and control (untreated cells, NT), which suggests that the naked siRNA could not inhibit the proliferation of MCF-7 cells. On the contrary, the cell viability when treated with EP-ICONs was significantly lower than those incubated in the presence of naked siRNA and in the untreated control groups (* *p* < 0.05) in the R range 10–40. This result demonstrates that EP-ICONs were able to promote the intracellular delivery of siSUR and to inhibit to some extent the proliferation of MCF-7 cells. Moreover, at the same R (10), cell viability in the presence of E-ICONs R10 showed a significant reduction when compared with P-ICONs at the same weight ratio.

**Figure 8:**
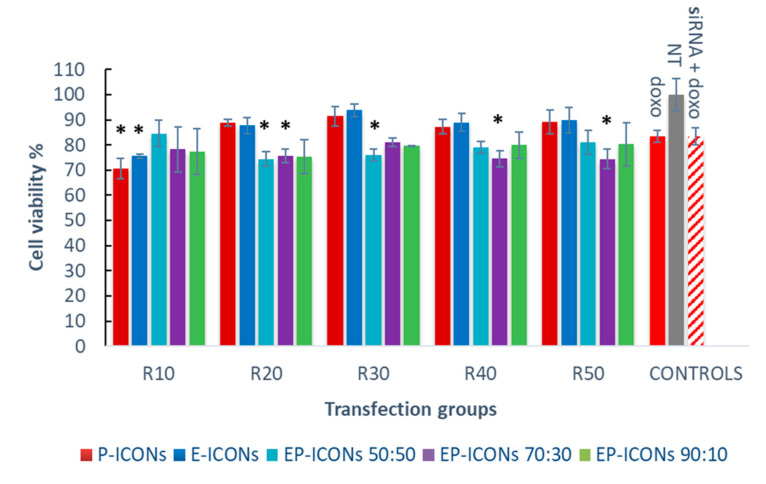
Chemosensitisation experiments. Effects on cell viability of Doxo (24 h) on MCF-7 cells after 24 h pre-treatment with E-ICONs, P-ICONs or EP-ICONs containing siSUR. * represents *p* < 0.05 with respect to cells treated with naked siSUR and Doxo (siRNA + Doxo).

Once we demonstrated the capability of EP-ICONs to allow the intracellular transport of loaded siSUR, we investigated the potential chemosensitisation effect of a combination therapy between siSUR (free or loaded into EP-ICONs) and a chemotherapeutic agent, such as doxorubicin (Doxo). The latter was selected as a model anticancer drug because its efficacy is often hampered due to the acquirement of drug resistance by cancer cells, and various evidence indicates that survivin inhibition enhances the antitumor activity of Doxo [18]. With this in mind, we studied the chemosensitisation effect on Doxo cytotoxicity promoted by 24 h pre-treatment of MCF-7 cells with siSUR, naked or loaded into EP-ICONs. The results are reported in Figure 8 as MCF-7 cell viability as a function of EP-ICONs, at a R ranging between 10 and 50.

It was found that while the pre-treatment with naked siSUR for 24 h before cell exposure to Doxo elicited no significant improvement in the drug cytotoxicity, a significant improvement in the Doxo cytotoxic effect was observed after 24 h of pre-treatment with EP-ICONS. In particular, the results demonstrate that the balance between EGF and PEG in EP-ICONs can modulate the EP-ICONs’ performance in producing a chemosensitisation effect on MCF-7 cells towards Doxo. Indeed, by using an INU-IMI-DETA-EGF/INU-IMI-DETA-PEG copolymer weight ratio of 50:50 and 70:30, EP-ICONs produced a significant chemosensitisation effect, which is up to a 10% reduction in cell viability with respect to cells treated with naked siSUR and Doxo (Doxo + siRNA control group). This effect is obtained at R ranging between 10 and 30 for an INU-IMI-DETA-EGF/INU-IMI-DETA-PEG copolymer weight ratio of 50:50, and at R between 20 and 50 for the polymeric mixture at 70:30.

In contrast, E-ICONs and EP-ICONs at an INU-IMI-DETA-EGF/INU-IMI-DETA-PEG copolymer weight ratio of 90:10 showed a behaviour comparable to E-ICONs. This effect can be explained by considering that in these samples, EGF could be directly involved in the polyplex formation between INU-IMI-DETA-EGF and siSUR, probably also due to the low PEG content. As reported elsewhere, a strong interaction between polycation and siRNA in one hand ensures high siRNA loading, stability, and protection, and on the other hand, it can represent an obstacle to efficient drug release once inside cells [26]

P-ICONs also did not produce a significant chemosensitisation effect on MCF-7. In effect, while pegylation could be useful in ameliorating circulation and colloidal stability, it often can produce (as demonstrated with uptake studies) a reduction in cellular uptake and endosomal escape, which results in significant loss of activity of the untargeted delivery nanosystems [27,28].

## 3. Materials and Methods

### 3.1. Materials

Inulin (INU) from dahlia tubers (Mw ≈ 5000 Da), diethylenetriamine (DETA) and 4-imidazoleacetic acid hydrochloride (IMIAc), bis(4-nitrophenyl) carbonate (4-BNPC), *N*,*N*′-dicyclohexylcarbodiimide (DCC), 4-(dimethylamino)pyridine (DMAP), dichloromethane (DCM), *N*-hydroxysuccinimide (NHS), *N*-(3-dimethylaminopropyl)-*N*′-ethyl carbodiimide hydrochloride (EDCI), epidermal growth factor human recombinant (hEGF, expressed in *E. Coli*), doxorubicin hydrochloride (Doxo) acetone, ethidium bromide, and ribonuclease A from bovine pancreas (RNase A) were purchased from Sigma Aldrich. Pullulan standards (in the range 180–47,300 Da) were purchased from Polymer Laboratories. Anhydrous *N*,*N*-dimethylformamide (a-DMF) was purchased from VWR. Anhydrous dimethylsulphoxide (a-DMSO) was purchased from Alfa Aesar (Kandel, Germany).

Duplexed siRNAs were purchased from Eurofins MWG operon (Ebersberg, Germany). The sequences (5′→3′) of sense strands are reported below:

Luciferase GL3 (siGL3): CUUACGCUGAGUACUUCGA(dTdT), with and without Cy5 linked to the 5′ end; survivin (siSUR): GCAUUCGUCCGGUUGCGCU(dTdT).

^1^H-NMR spectra were recorded in D_2_O (VWR) using a Bruker AC-250 spectrometer operating at 250.13 MHz (Bruker Corporation, Billerica, MA, USA).

FT-IR spectra were recorded on the solid sample in the frequency range of 4000–400 cm^−1^ by using a Bruker ALPHA FT-IR Spectrometer (Bruker Corporation, Billerica, MA, USA), equipped with Eco ATR single reflection sampling module with a ZnSe ATR crystal. Spectra were recorded in reflectance scale with a resolution of 1 cm^−1^ and 100 scans.

### 3.2. General Procedure for the Synthesis and Characterisation of Inulin-g-Imidazole (INU-IMI) and INU-g-IMI-g-Diethylenetriamine (INU-IMI-DETA) Copolymers

INU-IMI and INU-IMI-DETA copolymers were synthesized by the previously reported procedure [13]. The product characteristics were confirmed by ^1^H-NMR analysis in D_2_O/DCl. ^1^H-NMR of INU-IMI reveals peaks at δ: 3.7 ppm (m, 5H_Inu_, –CH_2_OH; –**CH**CH_2_OH; –C**CH_2_**O–), 3.9 ppm (t, 1H_Inu_, -**CH**OH), d 4.1 ppm (d, 1H_Inu_, -**CH**OH), 4.3 ppm (m, 2H_IMIAc_ -CHCH_2_O(CO)**CH_2_**C-), 7.3 ppm (m, 1H_IMIAc_, -CHCH_2_O(CO)CH_2_C=**CH-**), 8.6 ppm (m, 1H_IMIAc_, -CHCH_2_O(CO)CH_2_CN=**CH-**). ^1^H-NMR of INU-IMI-DETA reveals peaks at δ: 3.0–3.5 ppm (m, 4H_Deta_, -**CH_2_**NH**CH_2_-**; m, 2H_Deta_, -**CH_2_**NH_2_; m, 2H_Deta_, -OCONH**CH_2-_**), 3.7 ppm (m, 5H_Inu_, -**CH_2_**OH; -**CH**CH_2_OH; -C**CH_2_**O-), 3.9 ppm (t, 1H_Inu_, -**CH**OH), d 4.1 ppm (d, 1H_Inu_, -**CH**OH), 4.3 ppm (m, 2H_IMIAc_ -CHCH_2_O(CO)**CH_2_**C-), 7.3 ppm (m, 1H_IMIAc_, -CHCH_2_O(CO)CH_2_C=**CH-**), 8.6 ppm (m, 1H_IMIAc_, -CHCH_2_O(CO)CH_2_CN=**CH-**). Residual internal D_2_O (δ 4.8).

### 3.3. General Procedure for the Synthesis and Characterisation of INU-IMI-DETA-EGF Copolymer

INU-IMI-DETA (30 mg, 322.5 × 10^−4^ mmoles of DETA) were dispersed in 1 mL of bi-distilled water. Then, 250 µL of an NHS water solution (0.18 mg/mL, 3.225 × 10^−4^ mmoles of NHS), 250 µL of EDCI water solution (0.22 mg/mL, 3.870 × 10^−4^ mmoles of EDCI) and 1 mL of a hEGF water solution (2 mg/mL, 3.225 × 10^−4^ mmoles of hEGF) were added dropwise under constant stirring at room temperature to the INU-IMI-DETA dispersion. After 6 h, the mixture was diluted with bi-distilled water and dialysed exhaustively against bi-distilled water at 4 °C using a float-ALyzer G2 Dialysis Device (MWCO: 8–10 KDa). The solid product was obtained after freeze-drying. The presence of EGF in the polymeric structure was confirmed by FT-IR analysis. The degree of functionalisation in EGF was obtained indirectly by quantitation of thiol and disulphide groups by employing a method already described [19,29]. Briefly, DTNB, dissolved in 1M Na_2_SO_3_ pH 7.5, was converted into 2-nitro 5-thio sulfo benzoic acid (NTSB) by gurgling oxygen into the solution in a water bath at 38 °C until it was pale yellow (≈45′). Before use, NTSB solution was diluted 1:100 with 100 mM Na_2_SO_3_, 3 mM EDTA, 50 mM glycine buffer pH 9.5. INU-IMI-DETA-EGF solution (0.4 mL, 10 mg/mL) or cystamine standard solutions (0.4 mL, 0.00005–0.001 mmol/mL) in bi-distilled water were added to 2 mL of reaction buffer, then kept for 10 min in the dark and the absorbance was read at 410 nm in a plate reader (AF 2200, Thermofisher, Eppendorf Hamburg, Germany). The absorbance of a blank constituted by 0.4 mL of water and 2 mL of reaction buffer was also recorded as blank to calibrate the plate reader to zero absorbance.

### 3.4. General Procedure for the Synthesis and Characterisation of Inulin-g-Imidazole-g-Diethylenetriamine-g-PEG (INU-IMI-DETA-PEG) Copolymer

INU-IMI-DETA (100 mg, 0.38 mmoles of fructose repeating units) were dispersed in 5 mL of bi-distilled water. The pH of the obtained dispersion was adjusted to 5.0 and PEG_2000_CHO (38.4 mg, 0.019 mmoles) were added. The mixture was reacted overnight under continuous stirring at room temperature. Then, the mixture was diluted with 20 mL of bidistilled water and purified by unbounded PEG_2000_CHO by exhaustive dialysis against bi-distilled water, using a Spectrapor dialysis membrane 2000 MWCO. The solid product was obtained after freeze-drying. The product characteristics were confirmed by ^1^H-NMR analysis in D_2_O/DCl. ^1^H-NMR of INU-IMI-DETA-PEG reveals peaks at δ: 1.0–1.5 ppm (m, 4H_PEG_, -N=CH**CH_2_CH_2_**CH_2_CH_2_CONH-); 1.9–2.2 ppm (m, 4H_PEG_, -N=CHCH_2_CH_2_**CH_2_CH_2_**CONH-); 2.9–3.3 ppm (m, 4H_Deta_, -**CH_2_**NH**CH_2_-**; m, 2H_Deta_, -**CH_2_**NH_2_; m, 2H_Deta_, -OCONH**CH_2-_**), 3.3–3.7 ppm (m, 5H_Inu_, -**CH**OH; -**CH**CH_2_OH; -C**CH_2_**O-; s, 176H_PEG_, -[**CH_2_CH_2_**O]_44-_; m, 2H_IMIAc_ -CHCH_2_O(CO)**CH_2_**C-), 3.8 ppm (t, 1H_Inu_, -**CH**OH), d 4.0 ppm (d, 1H_Inu_, -**CH**OH), 6.5 ppm (m, 1H_PEG_, -N=**CH**CH_2_CH_2_CH_2_CH_2_CONH-); 7.1 ppm (m, 1H_IMIAc_, -CHCH_2_O(CO)CH_2_C=**CH**), 8.4 ppm (m, 1H_IMIAc_, -CHCH_2_O(CO)CH_2_CN=**CH-**). Residual internal D_2_O (δ 4.8).

### 3.5. Preparation of INU-IMI-DETA-EGF/PEG/siRNA “Complex Nanoaggregates” (EP-ICONs)

A proper volume of a siRNA water solution (0.025 µg/µL) was added by gently pipetting to the same volume of INU-IMI-DETA-EGF/INU-IMI-DETA-PEG solutions in water at pH 4 at various concentrations to obtain a defined copolymer/siRNA weight ratio ranging between 5 and 50. The relative composition by weight between INU-IMI-DETA-EGF and INU-IMI-DETA-PEG in the solutions were 100:0, 90:10, 70:30, 50:50, or 0:100. After at least 2 h, complexes were added to DPBS pH 7.4 (vol/vol ratio equal to 1/2). The obtained INU-IMI-DETA-EGF/INU-IMI-DETA-PEG complex nanoaggregates (EP-ICONs) were used without further purifications within one hour after preparation for each subsequent experiment.

### 3.6. Ethidium Bromide (EtBr) Exclusion Assay

Dose-dependent condensation and encapsulation efficiency of siRNA by EP-ICONs were examined by the quenching of EtBr fluorescence in an EtBr exclusion assay. To EP-ICONs, prepared as described above, 40 µL of EtBr in DPBS pH 7.4 (5 × 10^−3^ mg/mL) was added, and the samples were incubated in the dark for 15 min. After this time, the fluorescence of the samples was measured using a plate reader (AF 2200, Eppendorf Hamburg, Germany) at an excitation wavelength of 530 nm and an emission wavelength of 590 nm. The results were expressed as a percentage relative to the fluorescence of the naked siRNA-EtBr sample.

### 3.7. siRNA Stability in the Presence of RNase

To measure the ability of EP-ICONs to protect siRNA against nuclease degradation, the hyperchromic effect, i.e., the increase in absorbance at 260 nm that occurs when nucleic acids degrade, was used as previously described [28,30]. To 120 µL of EP-ICONs, prepared at various R (5–50) as described above, was added 5 × 10^−3^ µg of RNase A (70 U/mg) in 80 µL DPBS. Enzymatic degradation was monitored for 24 h by measuring absorbance at 260 nm at various time intervals on a plate reader (AF 2200, Eppendorf Hamburg, Germany). The results were plotted as increment % of absorbance at 260 nm (Abs_260_) versus time (minutes). Each point of the obtained curves is presented as mean ± standard deviation for triplicated samples.

### 3.8. Size and ζ Potential Measurements

Dynamic light scattering studies (DLS) were performed in DPBS at 25 °C with a Malvern Zetasizer Nano ZSP instrument fitted with a 532 nm laser at a fixed scattering angle of 173°, using the Dispersion Technology Software 7.02. The intensity-average hydrodynamic diameter (nm), and polydispersity index (PDI) were obtained by cumulative analysis of the correlation function. Zeta potential measurements were performed by aqueous electrophoresis measurements, recorded at 25 °C using the same apparatus. The zeta potential values (mV) were calculated from the eletrophoretic mobility using the Smoluchowsky relationship.

### 3.9. Biological Characterisation

#### 3.9.1. Hemocompatibility

Hemocompatibility was assayed by microscopic visualisation of human red blood cells (hRBCs) by an Axio Vert.A1 fluorescence microscope (Zeiss, Milan, Italy) equipped with an Axio Cam MRm (Zeiss), as previously reported [29]. Moreover, haemolysis assay according to [29] was performed. hRBCs isolated from fresh anticoagulant-treated blood were collected by centrifugation at 2200 rpm for 10 min at 4 °C. The pellet was washed with DPBS at pH 7.4 until the supernatant was clear by centrifugation and suspended in the same buffer. Afterwards, it was diluted in DPBS at pH 7.4 to a final concentration of 4 v% erythrocytes. This stock dispersion was used within 12 h after preparation. Two hundred microliters of INU-IMI-DETA-EGF or INU-IMI-DETA-PEG copolymer dispersions (in a concentration range between 25 and 1000 µg/mL) were added to the same volume of the hRBCs suspension and incubated for 1, 4, or 24 h at 37 °C under constant shaking. After centrifugation, the release of haemoglobin was determined by photometric analysis of the supernatant at 570 nm. Complete haemolysis was achieved by using a 1 v% aqueous solution of Triton X-100 (100% control value). Each experiment was performed in triplicate and repeated twice. The erythrocyte lysis percentage was calculated as % lysis = [(Abs_sample_ − Abs_blank_)/(Abs_100% lysis_ − Abs_blank_) × 100], where Abs_sample_ is the absorbance value of the haemoglobin released from erythrocytes treated with INU-IMI-DETA-EGF or INU-IMI-DETA-PEG copolymers, Abs_blank_ is the absorbance value of the haemoglobin released from erythrocytes treated with DPBS buffer, and Abs_100% lysis_ is the absorbance value of the haemoglobin released from erythrocytes treated with 1 v% aqueous solution of Triton X-100. The results represent mean ± standard deviation.

#### 3.9.2. Cell Cultures

Biological evaluations were conducted on human breast carcinoma cell line (MCF-7) and human bronchial epithelial cell line (16-HBE) purchased at the Istituto Zooprofilattico Sperimentale della Lombardia e dell’Emilia Romagna). MCF-7 cells were grown in Dulbecco’s modified Eagle’s medium (DMEM) with 10% foetal bovine serum (FBS), 1% of glutamine and 1% of penicillin/streptomycin (100 U/mL penicillin and 100 mg/mL streptomycin), at 37 °C in 5% CO_2_ humidified atmosphere. DMEM and the other cell culture constituents were purchased from Euroclone.

#### 3.9.3. Cell Viability Assay

MCF-7 or 16HBE cells were seeded in a 96-well plate at a density of 2 × 10^4^ cells/well. After 24 h, DMEM was replaced with 200 μL of OPTI-MEM medium containing INU-IMI-DETA-EGF or INU-IMI-DETA-PEG graft copolymer at various concentrations ranging from 25 to 1 × 10^3^ µg/mL, or siSUR-loaded EP-ICONs at different R, while cells treated with 200 μL of fresh OPTI-MEM medium were used as a control with 100% viability. After 72 h of incubation, cells were washed with 100 µL of sterile DPBS and incubated with fresh DMEM containing 20 v% of MTS reagent solution (3-(4,5dimethylthiazol-2-yl)-5-(3-carboxymethoxyphenyl)-2-(4-sulfophenyl)-2*H*-tetrazolium). Plates were incubated at 37 °C for 2 h, and after this time, the absorbance of formazan was measured by a UV plate reader, at 492 nm. Wells filled with MTS reagents at the same concentration in DMEM were used as blank to calibrate the spectrophotometer to zero absorbance. The cell viability values (%) compared to control cells were calculated by [(Abs sample/Abs control) × 100] and represent mean ± standard deviation for triplicated samples.

#### 3.9.4. Cell Uptake

MCF-7 cells were seeded in a 24-well plate at a density of 1.2 × 10^5^ cells/well. After 24 h, the culture medium was replaced by 600 µL of OPTI-MEM I medium containing siGL3-Cy5 loaded EP-ICONs at different copolymer/siRNA weight ratios, to reach a final siRNA concentration of 200 nM. After 4 and 24 h incubation, cells were extensively washed with sterile DPBS and lysed in 100 µL lysis buffer (2% SDS, 1% Triton X-100, in sterile DPBS). The lysates were divided into two parts: the first one (75 µL) was used to measure the fluorescence intensity by a Shimadzu RF-5301 PC spectrofluorophotometer (λ_ex_: 647 nm; λ_em_: 673 nm) calibrated with standard solutions of siGL3-Cy5 at various concentration ranging from 10 to 1000 nM in lysis buffer; the second one (25 µL) was used to evaluate the total protein amount by BCA protein assay. The results were expressed as the ratio ng of siRNA per milligram of protein and represent mean ± standard deviation for triplicated samples.

#### 3.9.5. Chemosensitivity Test

MCF-7 cells were seeded into 96-well plates (2.5 × 10^4^ cells/well) and left to adhere for 24 h. Cells were treated with siSUR-loaded EP-ICONs in OPTI-MEM or naked siSUR, at a final siRNA concentration of 200 nM, for further 24 h. After this time, cells were exposed to Doxo at a concentration of 2.5 µM in DMEM for 24 h. Finally, cell viability was detected by MTS as reported above.

### 3.10. Statistical Analysis

All experiments were conducted in triplicate. Quantitative data were expressed as the mean ± standard deviation. Statistical analysis was performed with Student’s *t*-test to evaluate the differences between groups. It was considered statistically significant when *p* < 0.05.

## 4. Conclusions

In this paper, “inulin complex nanoaggregates” (ICONs) with improved biological performance were obtained thanks to the chemical conjugation of EGF or PEG on the starting INU-IMU-DETA copolymer backbone. The unconventional choice to synthesize two INU-IMI-DETA copolymer derivatives bearing two different functions, one targeting and one that could confer stealth properties, was dictated by a precise desire to suitably modulate the composition of the resulting polymeric carrier according to the gene material to be conveyed. Therefore, the use of a mixture of INU-IMI-DETA-EGF and INU-IMI-DETA-PEG graft copolymers, from 0 to 100 wt% of each to produce complexes/nanoaggregates with siRNA, was revealed as a valid strategy to realise EGF-targeted pegylated ICONs (EP-ICONs). The latter were able to complex a higher amount of siRNA and to protect it from degradation by RNase, as compared to ICONs. Moreover, by in vitro experiments, it was demonstrated that the composition of EP-ICONs significantly affects the intracellular uptake of loaded siRNA, such as siSUR, and determine a significantly increase in cell chemosensitisation towards doxorubicin. It was evidenced how the fine tuning of the components can alter the behaviour in biological environment, showing that both EGF and PEG ligands need to improve the in vitro EP-ICONs performance. The sample obtained with a mixture of INU-IMI-DETA-EGF/INU-IMI-DETA-PEG at a weight ratio of about 70:30 was the best compromise in terms of chemical-physical properties and stability towards RNase, intracellular transport and activity of loaded siRNA, and chemosensitisation towards an antitumoral agent. Our study also gives new evidence of the ability of EGF conjugation to positively affect the retention of the siRNA. The balance of the two components, PEG and EGF, was shown to be fundamental, while confirming ICONs as a promising platform for siRNA smart delivery into tumours.

## Figures and Tables

**Table 1:** Composition of inulin complex nanoaggregates (ICONs) in terms of µmol/mg% of polyethylenglycol (PEG) and/or epidermal growth factor (EGF).

	PEG µmol/mg%	EGF µmol/mg%
E-ICONs	/	1.53 ± 0.17
EP-ICONs 90:10	1.12 ± 0.17	1.37 ± 0.15
EP-ICONs 70:30	3.36 ± 0.52	1.07 ± 0.12
EP-ICONs 50:50	5.61 ± 0.86	0.76 ± 0.08
P-ICONs	11.22 ± 1.73	/

## Data Availability

The data presented in this study are available in supplementary material.

## Supplementary Materials

The following are available online. Figure S1: ATR FTIR of INU-IMI-DETA and INU-IMI-DETA-EGF, Figure S2: Representative ^1^H-NMR of INU-IMI-DETA, INU-IMI-DETA-EGF and INU-IMI-DETA-PEG, Figure S3: Emocompatibility of INU-IMI-DETA and INU-IMI-DETA-PEG, Figure S4: Cell viability of 16HBE and MCF-7 cells after 72 h incubation with INU-IMI-DETA-PEG and INU-IMI-DETA-EGF, Figure S5: RNase protection assay on (A) P-ICONs, (B) E-ICONs, (C) EP-ICONs 90:10, (D) EP-ICONs 70:30, (E) EP-ICONs 50:50.

## Sample Availability

Not available.
