# Supplementary material for: Development of New Targeted Inulin Complex Nanoaggregates for siRNA Delivery in Antitumor Therapy
Source: Molecules. 2021 Mar 19;26(6):1713. doi: 10.3390/molecules26061713 (PMC8003534; doi:10.3390/molecules26061713)
Supplement: Supplementary file 1 [file molecules-26-01713-s001.pdf]

Supplementary Materials

Development of new targeted inulin complex nanoaggregates for siRNA delivery in antitumor therapy

Gennara Cavallaro*, Carla Sardo, Emanuela F. Craparo, Gaetano Giammona

Lab of Biocompatible Polymers, Department of Biological, Chemical and Pharmaceutical Sciences and Technologies (STEBICEF), University of Palermo, via Archirafi 32, Palermo 90123, Italy.

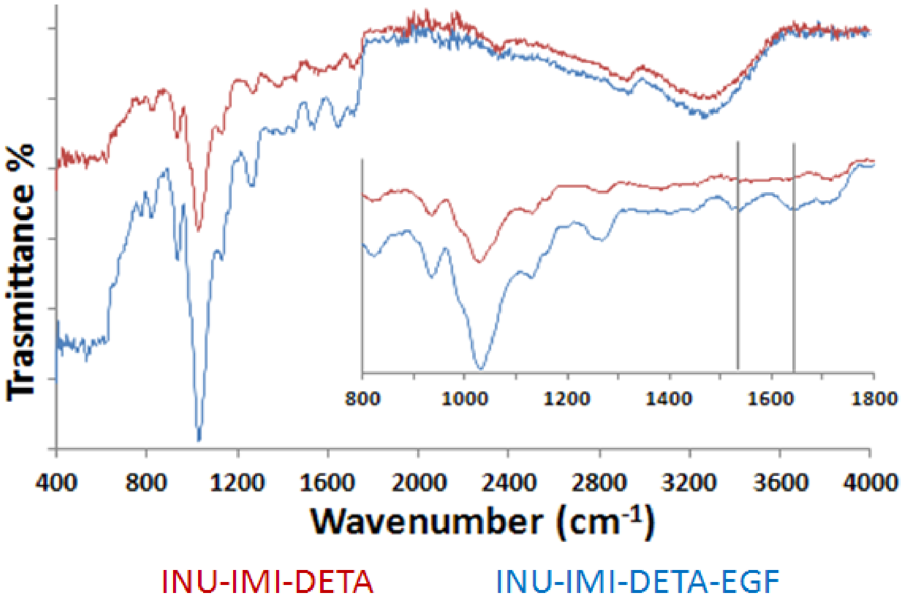

Figure. S1. ATR FTIR of INU-IMI-DETA and INU-IMI-DETA-EGF.

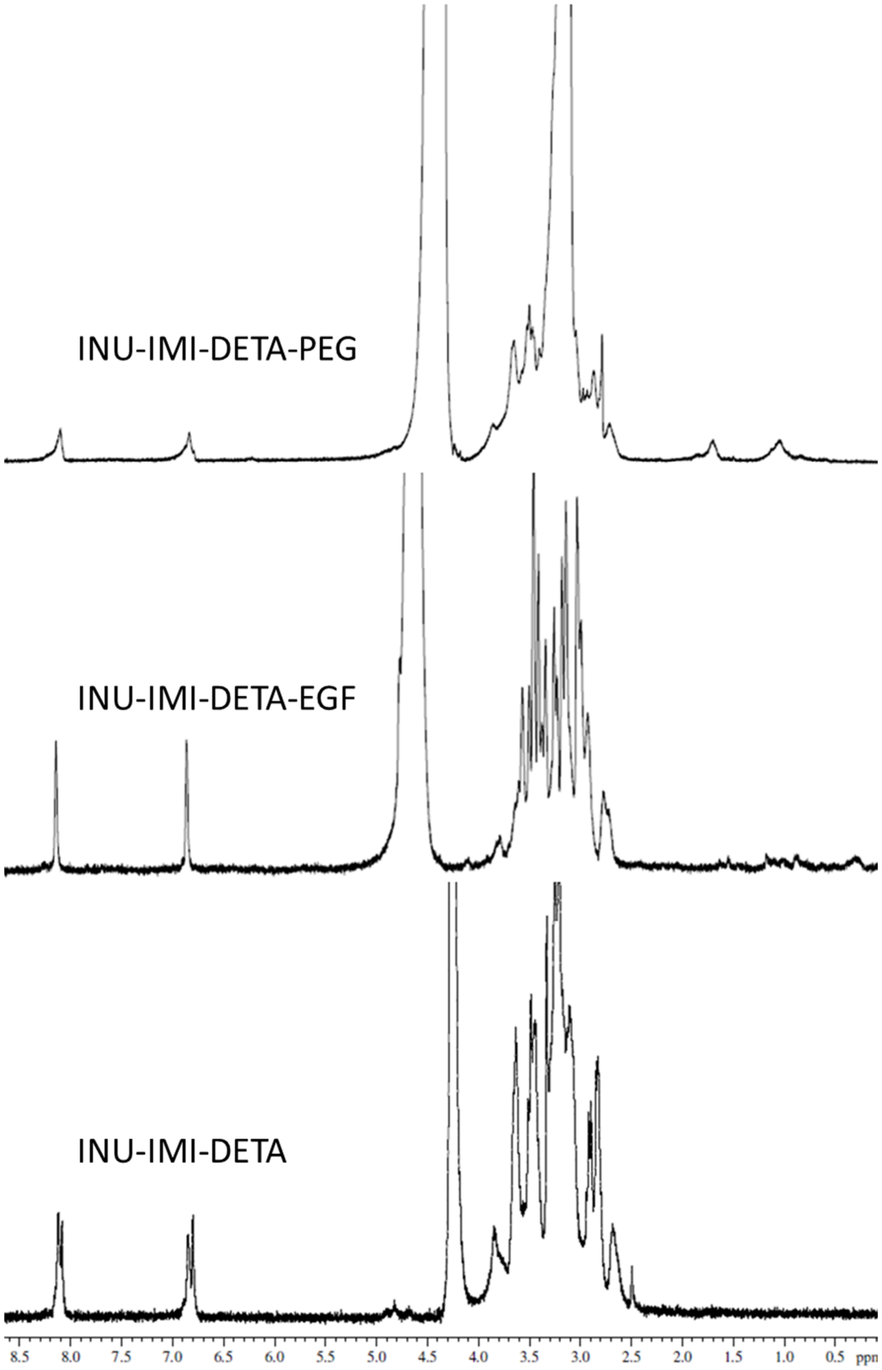

Figure. S2. Representative ^{1}H NMR of INU-IMI-DETA, INU-IMI-DETA-EGF and INU-IMI-DETA-PEG.

Haemocompatibility. Human red blood cells (hRBC) were used for assessing agglutination and hemolysis. These phenomena result from different interactions between RBC and copolymers. The former is due to simple surface interaction between positively charged polymeric unimers and negatively charged RBC membranes, whereas the latter, haemolysis, involves penetration and rupturing of the lipid bilayers triggered by surface interaction between positively charged self-aggregates or unimers and negatively charged RBC membranes.[1] Based on the experimental results (Figure S3), at pH 7.4 neither haemolysis nor agglutination of RBC occurred in the presence of the copolymers. This study demonstrates that even if INU-IMI-DETA and its derivatives destabilize the endosome/lysosome compartments through the proton sponge effect and destabilize the vesicle's lipid bilayer through ionic interactions in an acidic environment, these substances are safe and do not produce the above-mentioned effects when pH is 7.4.

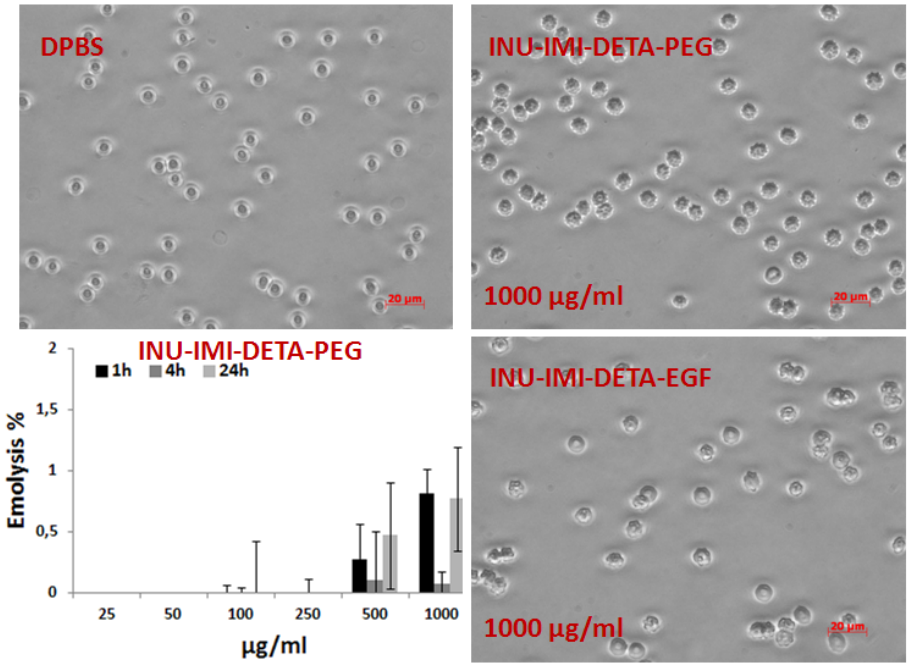

Figure S3. Emocompatibility of INU-IMI-DETA and INU-IMI-DETA-PEG.

Cell viability. Incubation effects of INU-IMI-DETA-EGF and INU-IMI-DETA-PEG were tested by MTS assay on human bronchial epithelial non tumoral cells (16HBE) and human breast adenocarcinoma (MCF-7) cells, at a range of concentration between 25 to 1000 µg/ml. 72 h

incubation with the copolymer didn't reveal any cytotoxic effect on both these two cell lines. The preliminary in vitro results justify the progression of the study towards the investigation of the potential use of such copolymers to construct siRNA delivery vehicles.

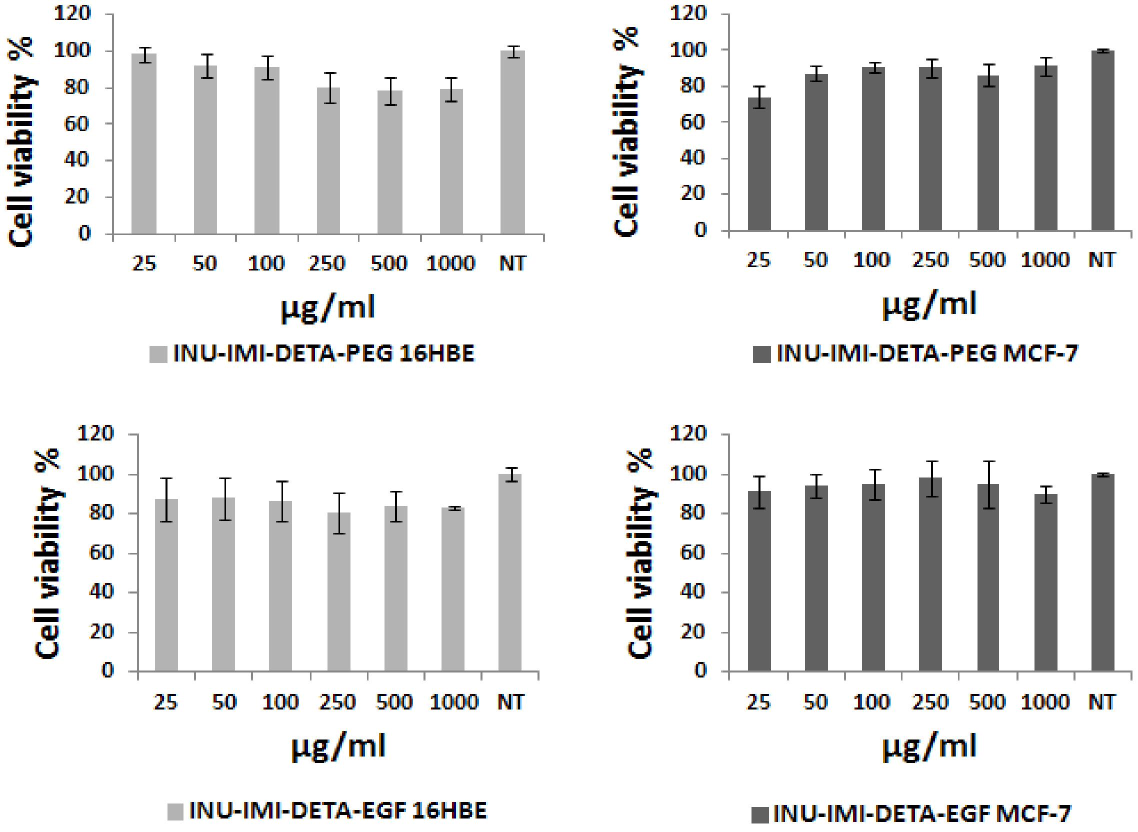

Figure S4. Cell viability of 16HBE and MCF-7 cells after72 h incubation with INU-IMI-DETA-PEG and INU-IMI-DETA-EGF.

RNase protection assay. When in contact with RNAse, PEGhylated systems offered the highest protection against siRNA degradation even at the lowest copolymer/siRNA weight ratio tested. As reported before by us, the percentual increment absorbance at 260 nm produced by ICONs (R5) after the first 20 minutes treatment with RNAse was equal to 7.56 ± 1.56 % (siRNA Abs 260 nm increment % after 20 minutes ≈ 19 %). At the same weight ratio tested, Abs 260 nm increment % equal to 2.37 ± 1.77 and 4.11 ± 2.16 for P-ICONs (R5), E-ICONs (R5) were found respectively.

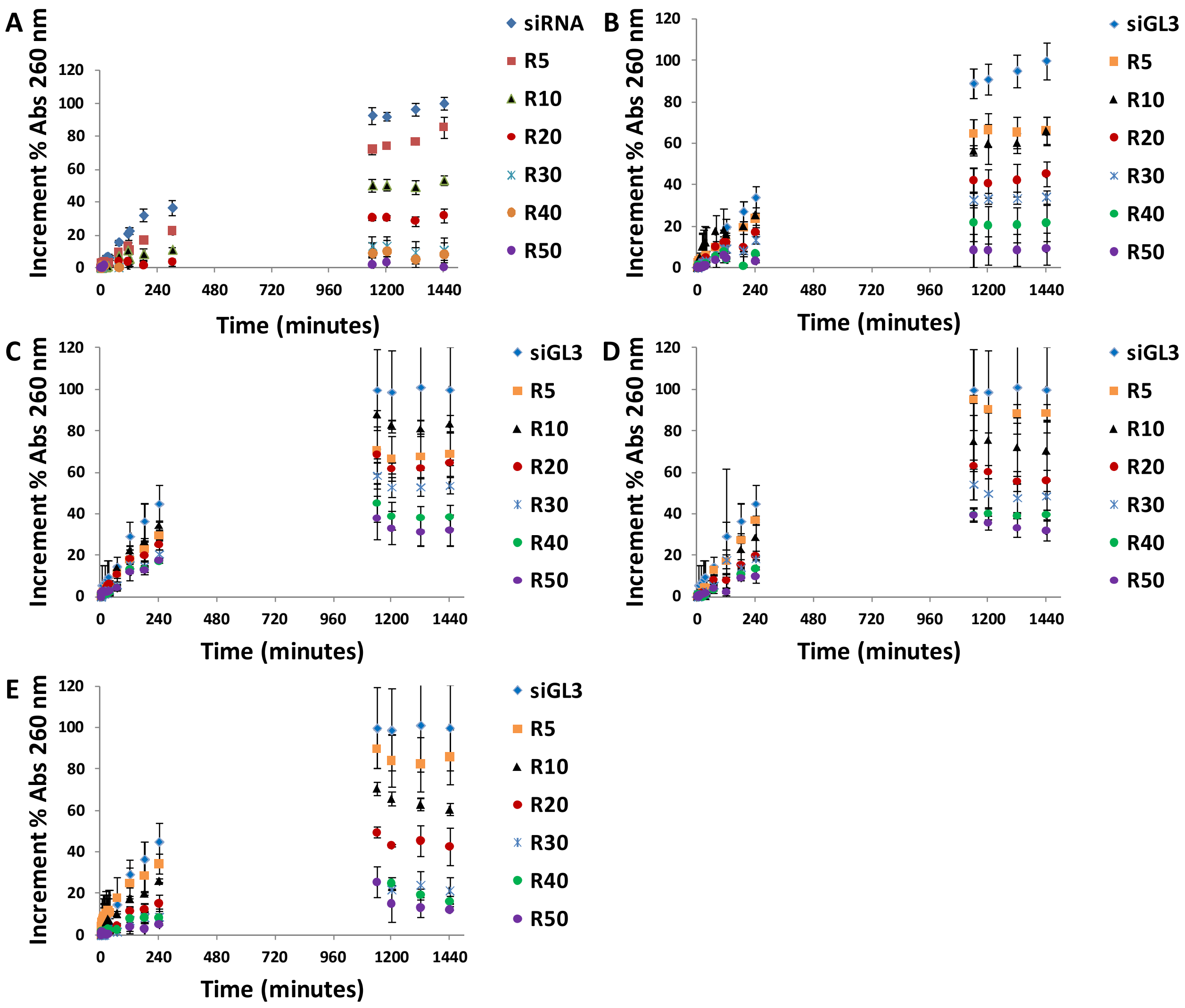

Figure S5. RNase protection assay on A) P-ICONs, B) E-ICONs, C) EP-ICONs 90:10, D) EP-ICONs 70:30, E) EP-ICONs 50:50.

References

[1] K. Seo, J.-D. Kim, D. Kim, pH-dependent self-assembling behavior of imidazole-containing polyaspartamide derivatives., J. Biomed. Mater. Res. A. 90 (2009) 478–86. doi:10.1002/jbm.a.32086.
